# Content Volatility of Scientific Topics in Wikipedia: A Cautionary Tale

**DOI:** 10.1371/journal.pone.0134454

**Published:** 2015-08-14

**Authors:** Adam M. Wilson, Gene E. Likens

**Affiliations:** 1 Geography Department, University at Buffalo, Buffalo, NY, United States of America; 2 Department of Ecology and Evolutionary Biology, University of Connecticut, Storrs, Connecticut, United States of America; 3 Cary Institute of Ecosystem Studies, Millbrook, New York, United States of America; University of Cape Town, SOUTH AFRICA

## Abstract

Wikipedia has quickly become one of the most frequently accessed encyclopedic references, despite the ease with which content can be changed and the potential for ‘edit wars’ surrounding controversial topics. Little is known about how this potential for controversy affects the accuracy and stability of information on scientific topics, especially those with associated political controversy. Here we present an analysis of the Wikipedia edit histories for seven scientific articles and show that topics we consider politically but not scientifically “controversial” (such as evolution and global warming) experience more frequent edits with more words changed per day than pages we consider “noncontroversial” (such as the standard model in physics or heliocentrism). For example, over the period we analyzed, the global warming page was edited on average (geometric mean ±SD) 1.9±2.7 times resulting in 110.9±10.3 words changed per day, while the standard model in physics was only edited 0.2±1.4 times resulting in 9.4±5.0 words changed per day. The high rate of change observed in these pages makes it difficult for experts to monitor accuracy and contribute time-consuming corrections, to the possible detriment of scientific accuracy. As our society turns to Wikipedia as a primary source of scientific information, it is vital we read it critically and with the understanding that the content is dynamic and vulnerable to vandalism and other shenanigans.

## Introduction

Wikipedia.org is now over ten years old and has become the sixth most popular website globally[1]. As of August 2014, over thirty thousand active editors were making three million edits per month to maintain and add topics to the four million articles in the English language version[2]. The Wikipedia infrastructure has made it possible for contributors to assemble quickly the world's largest and most up-to-date encyclopedia; the project has, in fact, been praised for accuracy that is 'close' to traditional reference sources such as the *Encyclopedia Britannica* [3–7]. There is some evidence that as Wikipedia matures, its scientific content increasingly references articles in established scientific journals and that the citation frequency of those journals is in general agreement with citation patterns in the scientific literature [8]. Scientists and students have also begun to embrace Wikipedia as a resource for public engagement and to improve scientific literacy[9]. For example, Bond argues that because Wikipedia has become such an important reference for so many people (ranging from students to scientists), that ornithologists (and other scientists) should ‘embrace and contribute’ to its content as an outlet for “science education, science communication, and public service” [10]. Despite these encouraging signs, however, there is reason to be concerned about the dynamic information content in Wikipedia.

One of Wikipedia’s fundamental principles is the “bold, revert, discuss” cycle, in which volunteer editors are encouraged to make ‘bold’ edits including adding, removing, rearranging, or rewriting the content in the article [11]. When someone else is monitoring the article, they are welcome to ‘revert’ any edit and return the article to its previous form. Following the reversion, the editors are encouraged to discuss the topic on the “talk” page that accompanies each article. In some ways, this mirrors the scientific process of peer review in which peers must critically evaluate new ideas, with the important exceptions that publication precedes review, there is no outright option for “permanent” rejection, and that the motivation, commitment, and qualifications of Wikipedia’s editors are typically unknown (especially for anonymous edits). To encourage participation, Wikipedia advises, “When in doubt, edit!” and then wait for someone else to revert your change. Neil Waters has written a related discussion of how the “democratization” of access to information is intertwined with the democratization of the information itself [12].

In addition to generating articles of unknown quality, this process can lead to 'edit-wars' between editors[13]. The problem is so severe for some articles that Wikipedia has guidelines to manage edit warring, including the three revert rule, which prevents editors from reverting more than three times on the same page in a 24-hour period. In general, though, overt edit warring is thought to be relatively uncommon; Sumi, et. al reported that “less than 1% of articles” are likely to have serious conflict[14]. However, little is known about how this potential for controversy affects the accuracy and stability of content on scientific topics, especially those with associated political controversy.

Following a long-standing research interest and expertise in acid rain [15], we noticed that some corrections we or others made on the acid rain article had been changed by major edits to introduce (or re-introduce) balderdash and factual errors into the content [16]. An illustrative example of tempestuous edits to the English language Wikipedia acid rain entry begins on November 30, 2011 [17]. At 10:20am, an anonymous editor (identified only by an IP address), removed the introductory paragraph which defined acid rain and replaced it with a statement calling acid rain “a load of bullshit.” This change was quickly reverted, but the next day the paragraph was again deleted and replaced by “Acid rain is a popular term referring to the deposition of wet poo and cats.” Five minutes later this edit was reverted and repeated again, and then reverted again. The following day (December 2, 2011) another sentence was changed from “During the 1990s, research continued.” to “During the 1990s, research *on elfs* continued [emphasis added],” which remained for over seven hours. Later that day the sentence "AciD Rain [sic] killed bugs bunny” was briefly added. Fifteen minutes later the section title “Chemistry in cloud droplets” was changed to “Blowjobs.”

Hopefully readers accessing the article during those tumultuous edits would recognize the jabberwocky via tone and absurdity. Algorithms that automatically detect and revert malicious edits like those described above are available and are continuously improving [18], but will probably never identify all cases of vandalism. For example, less than a month later, the sentences, “Acid rain does not directly affect human health. The acid in the rainwater is too dilute to have direct adverse effects” were briefly changed to “Acid rain directly affect [sic] human health. The acid in the water is too concentrated to have indirect adverse effects [19].” Similar edits have been made nearly every day over the history of the page despite the fact that it has typically had “protected” status to prevent edits by anonymous users [20].

After looking through this history and feeling frustrated by attempts at correction that were later changed, we decided to look at the issue more systematically. Given the political controversy that has surrounded the issue of acid rain, in this study we explore whether the frequency and magnitude of edits to the acid rain article are typical of scientific topics in general, or limited to controversial topics [21].

## Material & Methods

We compared three topics we consider to be politically (though not scientifically) controversial (acid rain, global warming, and evolution) and four we consider to be politically uncontroversial (heliocentrism, general relativity, continental drift, and the standard model in physics). To quantify the comparison, we downloaded the complete revision history of each article from 2003-06-12 (when the most recent article, Heliocentrism, originated) through 2012-07-31 (the most recent full archive available when this analysis was run) using Wikipedia’s export API (http://www.mediawiki.org/wiki/API:Main_page). The data were obtained in compliance with Wikipedia's Terms and Conditions of Use (http://wikimediafoundation.org/wiki/Terms_of_Use), which allows sharing and adapting the data with attribution. We then calculated three metrics from each article’s history: 1) daily edit rate (excluding successive edits by the same user, n = 23,156), 2) mean edit size (the total number of words inserted, deleted, or changed) on days with at least one edit (n = 8,525), and 3) mean number of page ‘views’ per day (which includes requests by computer programs, only available after 2008-01-01). We also calculated two-tailed nonparametric rank-based multiple contrasts to estimate the significance of pairwise differences in edit rates and words changed for each article with no distributional assumptions about the data [22]. The data and code used to run this analysis are available at http://dx.doi.org/10.6084/m9.figshare.1397533.

## Results & Discussion

The geometric means (±SD) ranged from 0.2±1.4 edits per day and 9.4±5.0 words changed per day for the standard model to 1.9±2.7 edits per day and 110.9±10.3 words changed per day for global warming (Table 1). The mean (±SD) number of page views ranged from 15 549±6 897 on the global warming page to 1 026 ± 564 on the heliocentrism page. We found that the edit rate of the acid rain article was less than the edit rate of the global warming (p<0.0001) and evolution (p<0.0001) articles, but significantly more (α = 0.05) than each of the ‘noncontroversial’ topics (Table 1). Furthermore, the three ‘controversial’ topics each had greater mean edit rates than each of the ‘noncontroversial’ topics (p<0.05). Similarly, the mean edit size for each ‘controversial’ topic was larger than each of the ‘non-controversial’ topics (p<0.05). While this analysis was intentionally limited in scope and it is difficult to assess causality (the “controversial” pages were also viewed more often, Table 1), edit rates can be much higher for “controversial” scientific topics. This finding is especially troubling in combination with the knowledge that Wikipedia content *about* scientists is not a good proxy for academic notability [23].

**Table 1:** Statistics summarizing the view and edit history of selected Wikipedia articles.

Wikipedia Article	Mean Daily Page Views ± SD^a^	Maximum daily edits^b^	Edits per day geometric mean ± SD (n) ^b^ ^,^ ^c^	Words changed per Day geometric mean±SD (n) ^b^ ^,^ ^d^
Acid_rain	2 954 ± 1 310	26	0.5±2.0 (3307)	36.2±10.2 (1103)
Global_warming	15 549 ± 6 897	231	1.9±2.7 (3307)	110.9±10.3 (2211)
Evolution	6 260 ± 2 450	89	1.3±2.5 (3307)	142.3±22.9 (1867)
Continental_drift	1 335 ± 641	19	0.3±1.7 (3307)	23.6±7.8 (844)
Heliocentrism	1 026 ± 564	20	0.3±1.6 (3307)	25.2±8.6 (818)
General_relativity	2 060 ± 1 443	37	0.4±1.7 (3307)	19.7±7.8 (1107)
Standard_model	1 202 ± 2 792	25	0.2±1.4 (3307)	9.4±5.0 (575)

^a^ “Mean Daily Page Views” from http://toolserver.org/~emw/wikistats/ were only available after 2008-01-01 and include programmatic page requests.

^b^ include data from 2003-06-12 (when the most recent article, Heliocentrism, originated) through 2012-07-31, when this analysis was run.

^c^ Mean daily edit count excludes successive edits by the same user (n = 23,156).

^d^ Mean count of words changed (inserted, deleted, or changed, n = 8,525). Due to the heavily right-skewed distributions, geometric means and standard deviations are shown. The number of observations (n) is constant for mean edits per day because all days were included, while only days with at least one edit were used to calculate the mean words changed.

So what should be done? In the future, it may be possible to automatically identify and flag pages with significant controversy [24] and quantify user reputation [18], both of which could be made visible to help readers critically evaluate the content of a page. For now, however, these results reinforce the position that Wikipedia should not be used in academic citations without very careful consideration and scrutiny [12]. Wikipedia acknowledges this and reports that, “while some articles are of the highest quality of scholarship, others are admittedly complete rubbish [25].” Furthermore, Wikipedia’s policy on academic use is clear that “Wikipedia is not considered a credible or authoritative source … any encyclopedia is a *starting point* for research, not an *ending point* [26].” What is needed is a wider appreciation of how to best leverage the vast quantity of information in Wikipedia to take advantage of its strengths (vast coverage and frequent updates) and avoid its weaknesses (potential for errors, conflict between editors, and content stability). Users should be aware that content in Wikipedia can be extremely dynamic; two students could obtain, within seconds, diametrically different information on a controversial scientific topic. Educators should ensure that students understand the limitations and appropriate uses of Wikipedia, especially for controversial scientific issues.
